# The Use of Platelet-Rich Plasma and Stem Cell Injections in Musculoskeletal Injuries

**DOI:** 10.7759/cureus.59970

**Published:** 2024-05-09

**Authors:** Nicole Schneider, Michael Sinnott, Nikita Patel, Roody Joseph

**Affiliations:** 1 Sports Medicine Department, Dr. Kiran C. Patel College of Osteopathic Medicine, Nova Southeastern University, Davie, USA; 2 Sports Medicine Department, Herbert Wertheim College of Medicine, Florida International University, Miami, USA

**Keywords:** platelet-rich plasma injections, musculoskeletal injuries, orthopedic procedures, musculoskeletal injections, stem cell injections

## Abstract

Injuries to the musculoskeletal (MSK) system can have a significant impact on an individual’s activities of daily living, as this multifunctional unit is associated with physical movement. Treatment of MSK injuries often involves corticosteroid injections, supplements, pharmaceutical agents, and/or surgery. While these approaches have been shown to be effective for some patients over both the short and long term, they can be associated with limited relief, adverse effects, and/or decreases in activities of daily living. An unmet need exists to develop and/or implement more effective treatment approaches for MSK injuries. Treatment options being explored include platelet-rich plasma (PRP) and stem cell injections. This review outlines the current state of research evaluating PRP and stem cell injections in the treatment of various MSK injuries. A literature search was conducted using the PubMed database to identify the relevant published articles related to the use of PRP and/or stem cell injections for the treatment of MSK and cartilage injuries. PRP and stem cell injections have been shown to improve an individual’s quality of life (QOL) and are associated with fewer side effects as compared to invasive standards of care in multiple MSK injuries such as plantar fasciitis, Achilles tendinopathy, acute muscle and tendon tears, ligament injuries, chondral and medial collateral ligament (MCL) knee injuries and arthritis, rotator cuff lesions, and avascular femoral necrosis. Specifically, these studies on PRP and stem cell injections suggest that both approaches are associated with a quicker return to activities of daily living while providing longer lasting relief without significant adverse events. The studies reviewed demonstrated PRP and stem cell approaches to be effective and safe for the treatment of certain MSK injuries, but as standardized protocols were not utilized across studies in the discussion of similar injuries, it was therefore difficult to compare their efficacy and safety. As such, further research is warranted to establish standardized research protocols across MSK injury studies to gain further insight into the efficacy, safety, and durability of PRP and stem cell injections.

## Introduction and background

The musculoskeletal (MSK) system is a dynamic and multifunctional unit of the human body that is composed of bones, cartilage, ligaments, connective tissue, and tendons, which function to support the body and aid in physical movement. As such, injuries to the MSK system, including acute muscle tears, plantar fasciitis, lateral ankle sprain, Achilles tendinopathy, knee injuries, and rotator cuff lesions, can be associated with significant impacts on an individual’s activities of daily living. 

Current standards of treatment for MSK injuries include the use of pharmaceutical agents, corticosteroid injections, supplements, and/or surgery. While these approaches have been shown to be effective for some patients over both the short and long term, they can be associated with limited relief, adverse effects, lack of regenerative benefit, and/or decreases in the activities of daily living [1]. Some potential complications include but are not limited to opiate addiction, organ failure induced by nonsteroidal anti-inflammatory drug (NSAIDs), limited long-term benefits of corticosteroid injections, and bleeding/reinjury associated with surgery [1].

Corticosteroid injections are presently used to treat multiple MSK conditions. Based on current research, steroid injections only remain efficacious for an average of three months and have significant side effects such as hyperpigmentation, infection, and post-steroid syndrome: flushing of the skin [2]. Research on pharmaceutical agents such as aspirin, acetaminophen, oxycodone, and other pain-modulating substances is significant. For instance, NSAIDs increase the incidence of renal and hepatic toxicities, gastrointestinal bleeds, and cardiovascular problems whereas opioid medications lead to increased incidence of constipation, mental addiction disorder, tolerance, respiratory depression, and death [3, 4]. These side-effect profiles limit the use of these drugs in clinical practice and thus pose a limitation to the patient population that can be treated, therefore opening an avenue to alternative pain-modulating therapies.

More effective approaches such as physical therapy and psychological treatments have been proven to be effective in providing long-term pain relief and improvements in overall quality of life (QOL) but may require longer time commitment [1]. As such, an unmet need exists to develop and/or implement more immediate treatment approaches for MSK injuries.

Treatment options being explored include platelet-rich plasma (PRP) and stem cell injections. This review outlines the current state of research evaluating PRP and stem cell injections in the treatment of various MSK injuries, suggesting that both approaches improve an individual’s QOL, maintain long-term efficacy, and are associated with fewer side effects as compared to standards of care in multiple MSK injuries such as plantar fasciitis, Achilles tendinopathy, acute muscle and tendon tears, ligament injuries, chondral and medial collateral ligament (MCL) knee injuries and arthritis, rotator cuff lesions, and avascular femoral necrosis [5].

**Figure 1:**
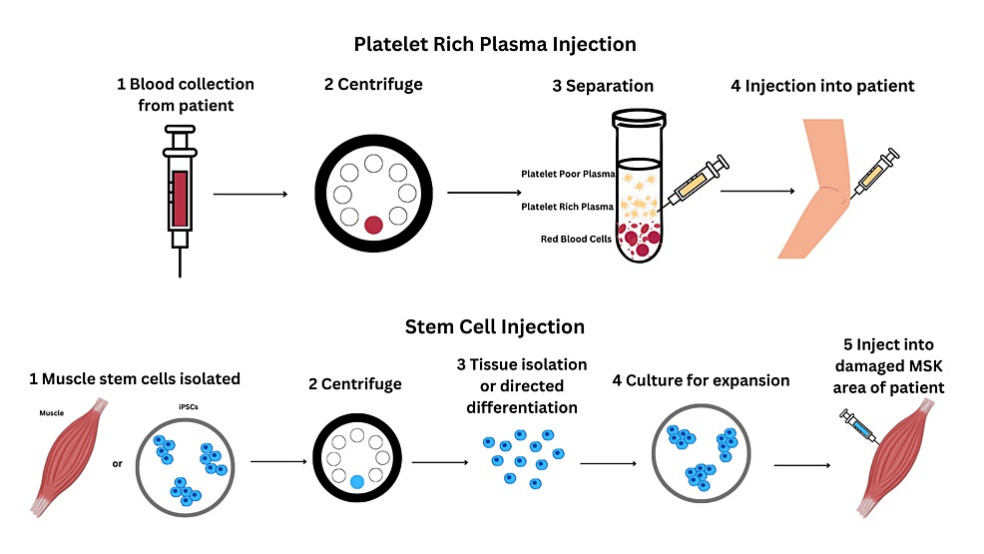
Scheme of platelet-rich plasma and stem cell injections For the stem cell injection, the isolated muscle stem cells were taken from the tissue or derived from the differentiation of induced pluripotent stem cells (iPSCs). Figure created using Canva and the data for the creation of the figure was taken from two articles [8,9]. MSK: musculoskeletal

Specifically, PRP’s purported effectiveness is derived from its growth factors such as platelet-derived growth factor (PDGF), transforming growth factor beta (TGFB), and insulin-like growth factors, and ability to increase blood supply and nutrients to the affected area [6, 7]. The process of the preparation of PRP injections is seen in Figure 1. PRP provides a minimally invasive avenue to restore joint function, mobility, and rebalance the body's homeostatic mechanisms by increasing type II collagen synthesis and vascularity, restoring hyaluronic acid, and reducing inflammation [7]. PRP also offers a minimally invasive approach to treat injuries while decreasing the potential side effects that current invasive modalities present for patients.

Meanwhile, stem cells are undifferentiated cells that differentiate into a particular cell lineage with a specific function upon reception of a growth stimulus. In a broad analysis, there are two stem cell types: embryonic stem cells (ESCs) and adult stem cells (Figure 1). Since ESCs are challenging to obtain and raise large ethical issues, adult stem cells have become the primary field of study, as these cells are more easily obtainable and exist in nearly all tissues [10]. Within the adult stem cell category, there are many cellular regulators that decide the fate of the cells, such as extracellular matrix molecules, growth factors, cytokines, and matrix stiffness [10]. The stem cells considered to be the most applicable are bone marrow-derived mesenchymal stem cells (bmMSCs), as they can be induced in vitro and in vivo and differentiate into multiple cell lineages, depending on which regulators influence them.

Invasive and pharmacologic treatments of MSK injuries have failed to provide lasting efficacy with minimal complication risk, demonstrating an unmet need for developing more effective management approaches [2]. Novel treatment modalities such as PRP and stem cell injections may result in an overall improvement of QOL, last longer than standard treatments, and present fewer adverse events with a potential for regeneration [5,10]. This review aims to provide an insight into the current studies using PRP and stem cells in treating MSK-related injuries, as well as discuss the effects that stem cells and PRP injections have on different MSK injuries throughout the body.

## Review

Study design

**Figure 2:**
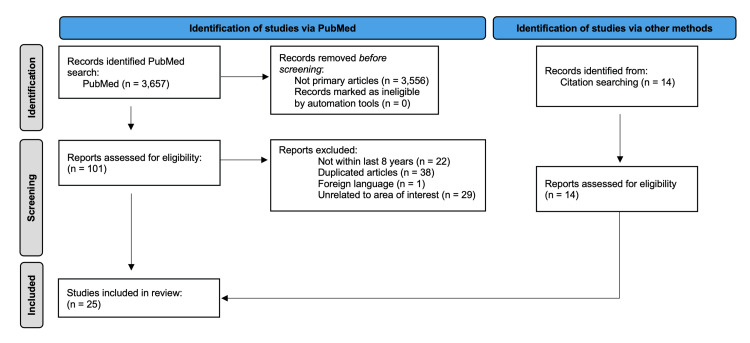
Flow diagram: identification of studies via PubMed and other methods

The articles discussed in this paper were gathered, analyzed, and evaluated using previously peer-reviewed published articles on the PubMed database. The keywords used to narrow the search included “Platelet-Rich Plasma AND Cartilage injuries", “Platelet-Rich Plasma AND Musculoskeletal injuries", “Stem Cell AND Cartilage injuries", “Stem Cell AND Musculoskeletal injuries”, “Platelet-Rich Plasma AND Musculoskeletal injuries AND Effectiveness AND Management”, “Stem Cell AND Musculoskeletal injuries AND Effectiveness AND Management”, “Platelet-Rich Plasma AND Cartilage injuries AND Effectiveness AND Management”, and “Stem Cell AND Cartilage injuries AND Effectiveness AND Management”. A total of 3,657 articles resulted, leading to a thorough review by applying the inclusion and exclusion criteria and removing any duplicates. The articles had to be primary articles, the time of their research within the last eight years, should have been published in English, should include cartilage injuries or MSK injuries using PRP and/or stem cell injections and exclude progenitor cells, platelet-rich fibrin, and any articles not including treatment of MSK and/or cartilage injuries with PRP and/or stem cells. Citation search was also included when conducting the review of PRP and stem cells. Therefore, a total of 25 articles resulted after the exclusion and inclusion criteria were applied. Eleven more articles were located on PubMed when conducting research on the background and discussion of MSK injuries and the current modalities used to treat these injuries other than PRP and stem cells as well as the creation of the figure. The information and clinical trials used in this review were limited to within the past eight years to better account for the newly emerging field of study and to provide the most accurate and up-to-date information. The identification of the articles was done and their eligibility for the review determined utilizing a flow diagram shown in Figure 2. 

Platelet-rich plasma

An unmet need exists in the management of musculoskeletal injuries to identify treatment options that are not only effective but associated with less adverse effects and faster return to activities of daily living and/or return to play. Currently, pharmacological therapies used for the treatment of musculoskeletal injuries have drawbacks and negative side effects including tolerance, addiction, and toxicity [1]. As such, alternative treatment approaches are much needed. PRP has demonstrated benefits without inducing the harmful side effects of currently available pharmaceutical agents and has been associated with a faster return to activities of daily living and/or playing sports. Specifically, PRP has been postulated to help increase tissue regeneration in a variety of injuries such as acute muscle tears, lumbar intradiscal disorders, plantar fasciitis, lateral ankle sprain, chronic midportion Achilles tendinopathy, acute Achilles tendon rupture, knee injuries, MCL injury, and rotator cuff lesions [11-20]. See Table 1 for platelet-rich-plasma-focused articles. 

**Table 1:** Summary points of platelet-rich plasma and stem cell articles PRP: platelet-rich plasma; QOL: quality of life; ROM: range of motion; bmMSCs: bone marrow mesenchymal stem cells; aMSCs: adipose-derived mesenchymal stem cells; MSCs: mesenchymal stem cells; OA: osteoarthritis; ON: osteonecrosis; BMAC-PRP: bone marrow aspirate concentrate-platelet-rich plasma [6,7,10,11-32]

Author(s), Year	Title	Sample Size	Results
Boesen et al. 2017 [6]	Effect of high-volume injection, platelet-rich plasma, and sham treatment in chronic midportion Achilles tendinopathy	57, male, 18- to 59-year-olds	PRP in combination with 12-week eccentric training allowed for a reduction in pain and tendon thickness, decreased intratendinous vascularity, and improved activity level.
Zou et al. 2020 [7]	Autologous platelet-rich plasma therapy for refractory pain after low-grade medial collateral ligament injury	52, male and female, 20- to 45-year-olds	PRP allowed for a reduction of pain, decreased tenderness and stiffness, complete restoration of function, no edema as well as shortened healing time. Alleviation of pain is possibly due to anti-inflammatory properties of PRP.
Li et al. 2015 [10]	A study of autologous stem cell therapy assisted regeneration of cartilage in avascular bone necrosis	15, male and female, 26- to 55-year-olds	Increased ROM and flexibility, restoration of femoral head cartilage, and decreased pain perception. However, the use of stem cell therapy is a subject of individual treatment.
Rossi et al. 2017 [11]	Does platelet-rich plasma decrease time to return to sports in acute muscle tear? A randomized controlled trial	72, male and female, 18- to 40-year-olds	Use of PRP allowed for decreased off-time (faster return to play) and decreased pain severity scores. Recurrence rate was not statistically significant between PRP and control groups.
Tuakli-Wosornu et al. 2016 [12]	Lumbar intradiskal platelet-rich plasma injections: a prospective, double-blind, randomized controlled study	43, female, 45- to 65-year-olds	PRP group showed improvement in function, pain, patient satisfaction, and duration of relief. No complications were reported post-injection.
Peerbooms et al. 2019 [13]	Positive effect of platelet-rich plasma on pain in plantar fasciitis: a double-blind multicenter randomized controlled trial	82, female, 40- to 60-year-olds	PRP had a decrease in pain and disability scores. The PRP group continued to improve whereas the corticosteroid group initially improved and later declined.
Blanco-Rivera et al. 2021 [14]	Treatment of lateral ankle sprain with platelet-rich plasma: a randomized clinical study	21, female, 18 to 60-year-olds	PRP injection allowed for faster recovery time, less pain, and better functionality.
Kearney et al. 2021 [15]	Effect of platelet-rich plasma injection versus sham injection on tendon dysfunction in patients with chronic midportion Achilles tendinopathy: a randomized clinical trial	221, male and female, 40- to 60-year-olds	A single PRP injection did not yield a statistically significant difference compared to the sham group of dry needling. Swelling was a side effect but did not last long.
Boesen et al. 2020 [16]	Effect of platelet-rich plasma on nonsurgically treated acute Achilles tendon ruptures: a randomized, double-blinded prospective study	40, male, 18- to 60-year-olds	Treatment with multiple PRP injections had no significant variation in tendon length, healing, or improved function between control and experimental groups. Therefore, no improvement in functional or clinical outcomes was seen, but different treatment regimens have yielded more successful outcomes.
Alsousou et al. 2019 [17]	Platelet-rich plasma injection for adults with acute Achilles tendon rupture: the PATH-2 RCT	216, male and female, 35- to 55-year-olds	PRP showed no significant variation between the control and experimental groups.
Danieli et al. 2021 [18]	Leucocyte-poor-platelet-rich plasma intraoperative injection in chondral knee injuries improved patient outcomes: a prospective randomized trial	64, male and female, 18- to 50-year-olds	Injection of PRP increased joint homeostasis and quality of synovial fluid as well as decreased synovial tissue inflammation. PRP can also slow down joint tissue degeneration.
Sari et al. 2020 [19]	Comparison of ultrasound-guided platelet-rich plasma, prolotherapy, and corticosteroid injections in rotator cuff lesions	120, male and female, 18- to 75-year-olds	A single dose of PRP showed a significant decrease in pain score post 24 weeks and long-term improvement in rotator cuff lesions.
Randelli et al. 2022 [20]	Platelet-rich plasma in arthroscopic rotator cuff repair: clinical and radiological results of a prospective randomized controlled trial study at 10-year follow-up	53 male and female, 54 to 67-year-olds	The PRP group reported less pain after two years; however, the difference between the PRP and control groups became negligible after 10 years.
Alsousou et al. 2017 [21]	Platelet-rich plasma in Achilles tendon healing 2 (PATH-2) trial: protocol for a multicenter, participant and assessor-blinded, parallel-group randomized clinical trial comparing platelet-rich plasma (PRP) injection versus placebo injection for Achilles tendon rupture	230, unspecified gender, 18-year-olds	PRP showed no significant variation between the control and experimental groups. This paper was the protocol for the study with the results reported in 2019, see Ref. [17].
Hamid et al. 2014 [22]	Platelet-rich plasma injections for the treatment of hamstring injuries: a randomized controlled trial	24, male and female, 17- to 49-year-olds	Patients with PRP injection along with the rehabilitation program recovered quicker within a short-term interval and reduced pain.
Ebert et al. 2017 [23]	A midterm evaluation of postoperative platelet-rich plasma injections on arthroscopic supraspinatus repair: a randomized controlled trial	55, male, 29- to 74-year-olds	Although no differences were observed between the PRP and control groups, it was noted that PRP did assist with improving pain-free abduction strength.
Hurd et al. 2020 [24]	Safety and efficacy of treating symptomatic, partial-thickness rotator cuff tears with fresh, uncultured, unmodified, autologous adipose-derived regenerative cells (UA-ADRCs) isolated at the point of care: a prospective, randomized, controlled first-in-human pilot study	15, male and female, 30- to 75-year-olds	The use of stem cells was safe without adverse side effects and improved the function of the shoulder.
Wyles et al. 2015 [25]	Adipose-derived mesenchymal stem cells are phenotypically superior for regeneration in the setting of osteonecrosis of the femoral head	15, male and female, 28- to 58-year-olds	aMSCs provided increased proliferation and bone differentiation efficiency compared to bmMSC in patients with osteonecrosis.
Alentorn-Geli et al. 2019 [26]	Effects of autologous adipose-derived regenerative stem cells administered at the time of anterior cruciate ligament reconstruction on knee function and graft healing	39, unspecified gender, 18- to 40-year-olds	aMSCs allowed for improvement in pain, knee function, activity level, and graft maturation; however, it was not statistically significant compared to the group without aMSC injection post reconstruction.
Kim et al. 2020 [27]	Implantation of mesenchymal stem cells in combination with allogenic cartilage improves cartilage regeneration and clinical outcomes in patients with concomitant high tibial osteotomy	70, male and female, 42- to 68-year-olds	Improvement in tibial/femoral axis deformity and in osteoarthritis grading. aMSC therapy provides symptomatic relief in cartilage-based repairs.
Hernigou et al. 2018 [28]	Subchondral stem cell therapy versus contralateral total knee arthroplasty for osteoarthritis following secondary osteonecrosis of the knee	30, male and female, 18- to 41-year-olds	Subchondral bone marrow injection was more effective compared to total knee arthroplasty for the treatment of OA in patients with ON due to corticosteroid use.
Toan et al. 2020 [29]	The effectiveness of knee osteoarthritis treatment by arthroscopic microfracture technique in combination with autologous bone marrow stem cell transplantation	46, male and female, 46- to 69-year-olds	Usage of autologous bone marrow stem cells was safe and effective in the treatment of OA due to a decrease in pain and improvement of knee function.
Rodas et al. 2021 [30]	Effect of autologous expanded bone marrow mesenchymal stem cells or leukocyte-poor platelet-rich plasma in chronic patellar tendinopathy (with gap >3 mm): preliminary outcomes after six months of a double-blind, randomized, prospective study	20, male, 18- to 48-year-olds	The use of bmMSC injection along with rehabilitation resulted in a reduction of pain, improved activity, and patellar tendon structure compared to PRP.
Kim et al. 2018 [31]	Effects of bone marrow aspirate concentrate and platelet-rich plasma on patients with partial tear of the rotator cuff tendon	24, male and female, 47- to 66-year-olds	BMAC-PRP injection improved shoulder function and pain. No significant difference in the decrease in tear size between the BMAC-PRP and control groups.
Kim et al. 2017 [32]	Effect of bone marrow aspirate concentrate-platelet-rich plasma on tendon-derived stem cells and rotator cuff tendon tear	1, female, 58-year-old	BMAC and PRP assisted with the healing of tendon tears, increased growth, and migration of tendon-derived stem cells.

Acute Muscle Tear with Platelet-Rich Plasma Injection

Researchers have demonstrated that PRP can positively affect growth factors to decrease the time of tissue regeneration in a variety of injuries such as tendon rupture, cartilage injury, and ligament sprains. Rossi et al. aimed to study the effects of PRP on return to play after acute muscle injury from recreational and competitive sports in athletes two years post injury [11]. This study focused on lower extremity muscle strains of hamstrings, gastrocnemius, and quadriceps by measuring four factors: (a) time to return to play from the date of injury to the day the full range of motion was achieved, (b) improvement of strength and functional abilities without pain/stiffness, (c) overall pain severity, and (d) recurrence rate of injuries/strains at two, 12, and 24 months. The researchers found that PRP had a significant impact on shortening the time to return to sports where the control group had a mean time of 25 days versus the PRP group having a mean time of 21.1 days. The recurrence rate at two years showed no statistical significance at 5.7% and 10% for the PRP group and control group, respectively [11]. This study was limited by the size of the study population and lack of statistically significant difference in visual analog score (VAS) scores and recurrence rate [11].

Lumbar Intradiscal with Platelet-Rich Plasma Injection

Low back pain is a significant cause of disability among Americans. Due to the intervertebral disc (IVD) being largely avascular, upon injury, healing can be problematic and may contribute to intervertebral disc syndrome (IDS). Given the failure of currently available pharmaceutical and other treatment modalities including surgery, PRP injections have been considered a potential treatment option for these patients, as PRP injections have been associated with increasing the amount of growth factors to promote the body’s natural healing processes [12]. PRP injections aim to offer a safer and more cost-effective approach as compared to surgery for patients with chronic lower back pain. In a study by Tuakli-Wosornu et al., two participant groups received either PRP injections (n=29) or a contrast agent (control; n=18) at the midportion of the suspected disc levels and an additional injection at the disc level that presented with pain [12]. The aim of this study was to measure the improvement in pain (using the numeric rating scale (NRS)), function (using the functional rating index (FRI)), degree of satisfaction (using the North American Spine Society (NASS) Outcome Questionnaire), and side effects including increased pain, bleeding, infections, and neurologic deficits over an eight-week period [12]. The researchers found that PRP injection into patients with chronic discogenic lower back pain resulted in greater improvement of function, less pain, and better satisfaction after eight weeks compared to the control group. As measured by the internationally validated outcome surveys (FRI, NRS, SF-36, and the Modified NASS Outcomes Questionnaire), pain and functional improvements showed a statistically significant p-value of .015 on the NRS scale for pain. In addition, these findings were sustained after one year. Further studies need to be performed to compare the control group with the experimental group after one year [12]. This study suggested that PRP therapy is promising for the long-term treatment of chronic low back pain with minimal side effects and warrants further investigation.

Plantar Fasciitis with Platelet-Rich Plasma Injection

Peerbooms et al. [13] utilized PRP injections on patients with chronic plantar fasciitis with the aim of decreasing pain and improving QOL. Two groups of patients were assessed in this study: one group in which patients received an injection of PRP and the other in which patients received an injection of corticosteroid. The Foot Function Index (FFI) pain score and World Health Organization Quality of Life Brief Version (WHOQOL-BREF) score were used at one, three, six, and 12 months post-injection therapy to measure the short-term and long-term effectiveness of these approaches as well as the presence of adverse events. After receipt of a PRP or corticosteroid injection, patients participated in stretching exercises prescribed by a physical therapist for two weeks, followed by strengthening exercises for two weeks [13]. At one year, PRP demonstrated improvement in patient’s QOL and decreased pain associated with chronic plantar fasciitis when compared to patients receiving corticosteroid injection [13]. This study suggests that PRP, in combination with directed stretching and strengthening exercises, is more effective than corticosteroid injections at one year due to the steady trend of pain relief, improvement of QOL, and decrease in risks associated with less repeat injections; however, future studies are warranted to determine long-term effects, adverse effects of the PRP injection, and comparison of PRP use alone without associated exercise.

Lateral Ankle Sprain with Platelet-Rich Plasma Injection

In 2019, a study by Blanco-Rivera et al. [14] evaluated the use of PRP in combination with short-term immobilization in patients diagnosed with acute lateral ankle sprains compared to a control group that underwent long-term immobilization. In this study, patients with lateral ankle sprains were injected with PRP followed by short-term immobilization for 10 days or only underwent long-term immobilization for three or more weeks. At eight-week follow-up, the PRP treatment group showed significant improvements in the VAS and the American Orthopedic Foot and Ankle Score (AOFAS) compared to the control group that underwent long-term immobilization [14]. Meanwhile, at 24 weeks follow-up, both groups reported comparable findings, VAS of 0.1 and 0.2 for the PRP and control groups, respectively (p-value=0.493) [14]. Based on these results, study participants who were administered the PRP injection adjuvant therapy experienced a faster recovery of pain, which enabled them to get back into their daily lives and sports faster than the control group at eight weeks follow-up. While PRP had no greater improvement in long-term (24 weeks) symptoms relative to long-term immobilization alone, utilizing PRP and short-term immobilization provides improved symptom scores (VAS scores) and may be considered an early treatment option. Further studies are warranted due to the small sample size of 21 participants (10 control, 11 treatment).

Chronic Midportion Achilles Tendinopathy with Platelet-Rich Plasma Injection

Achilles tendinopathy is commonly treated with injections, exercise, orthotics, and electrotherapy [15]. The use of PRP therapy in Achilles tendinopathy is thought to facilitate the repair of the tendon by increasing growth factors such as PDGF, TGFB, and insulin-like growth factor [6]. Boesen et al. [6] sought to evaluate this theory in their study comparing the effect of sham saline injections (control group) to PRP injections with eccentric exercise - provided by a physical therapist - in those with chronic Achilles tendinopathy (three months or greater) and the control group. Both groups were assessed for improvements in pain intensity, activity levels, tendon thickness, and tendon vascularity. The study results showed that multiple injections of PRP followed by a 12-week eccentric training program resulted in a reduction of pain, increased activity level, reduction of tendon thickness, and decreased intratendinous vascularity [6]. These findings suggest that PRP with exercise results in improved outcomes relative to sham treatments. The findings of this study support the need for further research on the effectiveness of exercise combined with PRP on chronic tendinopathy in comparison to the standard of care due to the limiting factor of being unable to determine the exact concentration of platelets in the PRP injections and lack of exercise protocol details. Therefore, a consistent protocol of platelet concentration and therapeutic exercise protocol will assist with supporting the suggested positive results of PRP injection on chronic tendinopathy.

Acute Achilles Tendon Rupture with Platelet-Rich Plasma Injection

Boesen et al. focused on the effect of multiple PRP injections to treat nonsurgically treated Achilles tendon rupture (ATR) compared to placebo injections. The purpose of this experiment was to determine the effects of multiple injections since previous studies found no differences in function and clinical outcomes of ATR PRP injections upon a singular dose [16]. Following at-home exercises for both the PRP and placebo group, pain and function were measured as well as Achilles tendon length (p-value >.05), calf circumference (p-value <.001), and ankle dorsiflexion range of motion (p-value <.001), all of which showed no significant variation between the control and experimental groups [16, 17, 21]. A standardized protocol of preparation of PRP injections is needed to better compare the results of single versus multiple injections across other similar studies in order to acquire more accurate data.

Knee Injuries with Platelet-Rich Plasma Injection

As chondral injuries have a decreased rate of healing and regeneration of fibrocartilage relative to hyaline cartilage, these injuries are difficult to treat and can result in decreased activity and early degeneration of the cartilage. The current treatment for chondral knee injuries is surgery, which leaves the cartilage more fragile, weaker, and with no prevention of degeneration due to the replacement of the cartilage with fibrocartilage [18]. PRP is currently being evaluated as an alternative treatment option. The regenerative properties of PRP including increased type II collagen synthesis, biological homeostatic regulator, hyaluronic acid restoration, reduction in inflammation, and induction of mesenchymal stem cell chondrogenesis can promote the healing process of chondral injuries and decrease pain from injuries such as tendinopathy and osteoarthritis [18]. Danieli et al. compared chondral knee injuries between two groups: a control group with no PRP injection and only surgery, and a treatment group with both surgery and PRP injection, by measuring improvement in function, joint homeostasis, synovial tissue inflammation, and quality of synovial fluid. The results of this study found that PRP treatment led to an improvement in function after anterior cruciate ligament (ACL) reconstruction and grade III chondral knee injury according to subjective International Knee Documentation Committee (IKDC) form, Knee Injury and Osteoarthritis Outcome Score (KOOS), and Tegner activity forms assessed before surgery and three, six, 12, and 24 months post-surgery [18]. PRP has the potential to improve joint homeostasis, decrease synovial tissue inflammation, and increase the quality of synovial fluid compared to surgery alone [18]. This is one of the few studies that suggest an improvement in the quality of synovial fluid with the use of PRP, but small participant size, lack of postoperative imaging, use of a single injection, and emphasis on small-medium size tears limit the study's generalizability.

Acute Hamstring Injury with Platelet-Rich Plasma Injection

Current treatment options most commonly used for acute hamstring injury include rest, ice, compression, and elevate (RICE), anti-inflammatory medications, rehabilitation programs, and electrotherapeutic treatments [22]. Hamid et al. evaluated the use of PRP for acute hamstring injury. Specifically, the researchers aimed to determine how a single PRP injection with a rehabilitation program would affect the return to play of athletes post hamstring injury compared to solely using a rehabilitation program. The measurements used were the number of days to return to play, pain severity, and pain intensity scores using Brief Pain Inventory - Short Form (BPI-SF), which also assisted with determining when the patient could return to the sport [22]. Patients also underwent an isokinetic strength assessment to determine hamstring strength. Patients with a single PRP injection along with a rehabilitation program recovered quicker than patients undergoing rehab alone without the use of PRP [22]. Hamid et al. focused on the short-term effects of PRP use in combination with a rehabilitation program, and whether the authors' findings remain true in the long term is an area for future study.

Medial Collateral Ligament Injury with Platelet-Rich Plasma Injection

One of the most common knee ligament injuries, MCL injury, has been shown to benefit from PRP. In one study, patients received three serial injections of PRP followed by a range of motion and progressive resistive weight-bearing exercises. The participants underwent follow-up at one, three, and six months, with comparisons made to their starting baseline IKDC reports [7]. The results suggest the use of PRP improved pain, stiffness, tenderness, gait stability, and walking with weight without pain as assessed by the IKDCb (p= 0.00). These findings are noteworthy because it shows that intra-articular injection of PRP for intractable pain after low-grade MCL injury was able to improve subjective reports of function up to six months post-treatment [7]. It is important to note that because this study did not have a control group, direct claims about the efficacy of PRP therapy is not possible, and the results warrant further investigation.

Rotator Cuff Lesions with Platelet-Rich Plasma Injection

Rotator cuff repairs usually involve surgical treatment, which may be associated with retears due to incomplete healing or from reinjuries. In one study, Sari and Eroglu compared three types of injections, corticosteroids, PRP, and prolotherapy, to a control of lidocaine, which were all injected into rotator cuff lesions [19]. Due to the varying methods of each type of injection, the authors could not arrive at a consensus about which injection was the most successful in healing these lesions. All four injections - corticosteroids, PRP, prolotherapy, and lidocaine - proved to be beneficial treatments according to the VAS, American Shoulder and Elbow Surgeons (ASES), and Western Ontario Rotator Cuff Index (WORC) scores [19]. Further studies are needed that involve a larger study sample and multiple PRP injections as well as a longer follow-up than just 24 weeks. In another study, Ebert et al. conducted the longest follow-up of arthroscopic supraspinatus rotator cuff repair along with two PRP injections at seven and 14 days after surgery [23]. Using the Oxford Shoulder Score (OSS), Quick Disabilities of the Arm, Shoulder, and Hand (Quick-DASH) questionnaire, and VAS for pain, it was discovered that no significant differences were observed between the PRP as an adjuvant to surgery and the surgery-only group. In conclusion, the PRP injection did aid in attaining pain-free abduction; however, no evidence was found for the PRP injection leading to a robust tendon repair [23]. In contrast, Hurd et al. found that PRP injection as an adjuvant to arthroscopic surgery of a rotator cuff lesion was beneficial, as it improved outcomes including decreased pain and increased healing [24]. Further research needs to be conducted on the treatment of rotator cuff lesions with PRP injections as an adjuvant to arthroscopic repair.

Arthroscopic Rotator Cuff Repair with Platelet-Rich Plasma Injection

Randelli et al. compared PRP injection to a control group of no injection after arthroscopic rotator cuff repair with a 10-year follow-up. It was noted that at the two-year mark, the PRP group reported less pain compared to the control group, but this difference subsided after 10 years [20]. A lack of a standardized PRP composition protocol makes it difficult to compare studies due to the various methods of PRP preparations. As such, evaluating the concentration of growth factors, cytokines, and other components within the PRP mixture as well as the location and number of the injections could help standardize PRP use in research for comparison to one another. There are limited studies following patients over 10 years, and therefore future research is needed to validate the outcome of PRP injections observed in this study over the long term.

Stem cells

Musculoskeletal surgery is tied to increased risk of infection, use of anesthesia, graft rejection, medication addiction and tolerance, and increased functional off-time. Researchers and clinical practitioners have been investigating the effectiveness of MSCs as an alternative solution by decreasing immune-mediated host-graft rejection through the utilization of the patient's own human leukocyte antigen (HLA) immunological markers. The application of MSCs looks to utilize a less invasive approach to regenerate new hyaline cartilage and bone by differentiating them into osteogenic and chondrogenic cells. There are different types of stem cells: embryonic and adult stem cells. Embryonic being difficult to harvest because they differentiate into any type of cell whereas adult stem cells are easier to extract but have a more limited differentiation potential [10]. The most adequate stem cells are MSCs derived from either bone marrow or adipose tissue, which can differentiate into a variety of tissue types [10, 25]. Stem cells have become a focus of study for MSK injuries due to the less invasive approach with possible less side effects than the current modalities. See Table 1 for a compilation of the stem cell-focused articles.

Avascular Necrosis of the Femoral Head with Stem Cell Injection

Li et al. focused on the use of MSCs to treat avascular necrosis (AVN) of the femoral head. The current treatment for AVN includes the drilling of holes into the femoral head in order to allow an increase in blood flow to this area of interruption of blood supply or replacement arthroplasty for more severe AVN injuries [10]. The need for a less invasive approach led to the study of cells as a method of treatment such as the usage of stem cells in early AVN of the femoral head. Stem cells hold the ability to allow for bone regeneration and cartilage repair and therefore become a potential treatment option for AVN. Following bmMSC extraction and injection at a three-month follow-up time point, patients showed improvements in pain, range of motion, flexibility, and restoration of femoral head cartilage compared to pre-surgery [10]. Utilizing the patient's own bmMSCs to regenerate cartilage avoids the introduction of foreign material, significantly reduces the rate of immune rejection, and optimizes ROM of the joint. This potentially decreases the duration of postoperative care and rehabilitation. Stem cell therapy may offer an alternative approach to treating AVN.

Bone Marrow MSCs Versus Adipose-derived MSCs with Stem Cell Injection

Wyles et al. suggest mSCs are promising cellular candidates for regenerative orthopedics. The use of bmMSCs has demonstrated limited efficacy due to high physiologic stress to the surrounding tissues, high cell turnover, and loss of potency with age and disease [25]. Meanwhile, adipose-derived MSCs (aMSCs) may be a more effective approach to regeneration as they are more protective against these factors. In one study comparing bmMSCs and aMSCs for the repair of osteonecrosis of the femoral head, aMSCs had higher levels of proliferation capacity and better bone differentiation efficiency, suggesting a better regenerative therapeutic strategy compared to bmMSCs [25]. This is one of the few studies that allows a comparison between two lineages of stem cells. Although this study shows that aMSCs hold more potential, future studies are needed to demonstrate long-term efficacy and to elucidate adverse event profiles.

Anterior Cruciate Ligament Tear with Stem Cell Injection

ACL tears are common knee injuries in soccer and other sports. ACL tears are most commonly treated with arthroscopic-assisted reconstruction, but there is an increase in off-time and imperfect repair [26]. Stem cells may be a method to circumvent surgical procedures. ACL reconstruction with intraoperative administration of aMSCs was compared to intraoperative treatment alone. Patients who received intraoperative administration of aMSCs after ACL reconstruction had no significant advantage over those patients who received treatment without aMSCs [26]. It is suggestive that aMSCs do not improve healing or shorten recovery time in patients after ACL reconstruction [26]. Conflicting results exist in the literature. Wyles et al. [25] and Kim et al. [27] suggest that aMSC therapy provides symptomatic relief in some injuries - for example, cartilage-based repairs - to a better extent than others, such as ligament injuries.

MSCs and Allogeneic Cartilage in Knee Osteoarthritis with Stem Cell Injection

Medial compartmental knee osteoarthritis (OA) is caused by uneven joint load in the knees causing cartilage damage and is a common problem that affects athletes and the general population, as individuals age. Current treatment involves surgical approaches including high tibial osteotomy (HTO), which shifts the contact pressure of the cartilage [27]. However, long-term effects of this approach are questionable, as the cartilage is not repaired. This technique allows patients to walk without pain, but degeneration of the articular surfaces can occur despite the HTO correction [27]. Cell-based tissue engineering such as MSCs shows promise as its aim is to repair the cartilage by filling in the hyaline cartilage-like substances that will not deteriorate. In addition, cartilage regeneration is likely associated with more natural motion of the joints and less pain when compared to an artificial joint. The aim of this study was to determine whether MSC implantation or MSC plus allogeneic cartilage (AC) implantation is effective in treating patients with unresolved radiographically diagnosed grade III or IV medial compartmental knee OA with tibial and femoral axis deformity. To monitor long-term and short-term effects, patients were evaluated clinically and radiologically before surgery and after surgery at four weeks, three months, six months, and one year [27]. Although the mechanism behind the cartilage regeneration is uncertain, it is believed that it is due to the cell-to-cell interaction between MSCs and chondrocytes; the implantation of MSCs with the mega cartilage allows for the regeneration of the cartilage [27]. These results are possibly due to the synergistic nature of the AC when working with MSCs allowing for improved integration into human cartilage tissues. While the studies proved promising, future studies should contain a control group that has HTO alone, allowing for a better comparison of efficacy between the two groups.

Osteoarthritis and Osteonecrosis of the Knee with Stem Cell Injection

Although total knee arthroplasty (TKA) is one of the most commonly performed surgeries to treat osteonecrosis (ON) in patients who have OA, new techniques such as bone marrow concentrate (BMC) injections aim to provide an alternatively better and more therapeutic treatment option. By stimulating the proliferation of progenitor cells using BMC, the therapeutic window of efficacy may be increased from a “standard” window of three years to at least a decade, providing an approach that can be beneficial to younger populations [28].

One study aimed to evaluate patients who underwent bilateral TKA while injecting one side with a subchondral injection of BMC. The study consisted of 30 patients who had secondary bilateral ON of the knees related to corticosteroids. Radiographs and MRI confirmed the collapse and degradation of the cartilage surface with secondary OA [28]. Mesenchymal stem cells were aspirated from the bone marrow and were centrifuged to collect the concentrate. Knee arthroplasty was performed and then MSCs were injected into the subchondral medial and lateral femorotibial compartments of each knee. Patients were then evaluated clinically between four and six weeks, three months, one year, and then annually for about 12 years. The study showed that TKA and injection with subchondral bone marrow was more effective when compared to TKA alone in treating OA in patients with ON related to corticosteroids [28]. Although the subjects were young, they tended to have lower activity compared to other patients of the same age due to their condition. In addition, no biopsies were performed on the cartilage, leading to a weaker understanding of its composition. Similar findings were reported by Toan et al. with regards to combining bone marrow stem cells with arthroscopy in order to improve the VAS score KOOS points, which deals with knee function and symptoms [29]. In this study, a fibrin scaffold was introduced during the arthroscopy to increase growth factors and cytokines via stem cells, which aided in the repair of the knee [29]. The method of combining bone marrow stem cells with arthroscopy led to an invasive approach that was both safe and effective.

Platelet-rich plasma and stem cells

PRP and stem cells have become new approaches to treating a variety of musculoskeletal injuries. As these two harvesting procedures, PRP and stem cells, are derived from autogenous grafts, there is a substantially decreased probability of drug-mediated toxicity, tolerance, immunogenic rejection, immunosuppression, and bleeding that are often associated with current modalities such as surgery, thus offering a different avenue of treatment for various musculoskeletal injuries. Although still considered an emerging field, the combination of PRP and stem cell treatment has been used to treat injuries including chronic patellar tendinopathy and rotator cuff tendon tears. See Table 1 for a list of platelet-rich plasma and stem cell-focused articles. 

MSCs and PRP in Chronic Patellar Tendinopathy

In one study, the researchers aimed to determine if PRP or bmMSC injections will help with pain, function, and tendon structure of a patellar tendinopathy along with a rehabilitation program at a six-month time point [30]. Two groups were evaluated: one group received bmMSC injections and the other group received PRP injections followed by both groups receiving the same supervised rehabilitation program. The results of this study indicated that following PRP or bmMSC injection therapy, there was a reduction of pain and improvement of activity in both groups; however, bmMSC showed greater improvement of tendon structure compared to the PRP [30]. Based on the results, the authors concluded that bmMSC offers a better therapeutic relief of patellar tendinopathy compared to PRP, leading to a faster recovery time and entry back into daily life. Further analysis and research are needed to determine the long-term benefits of PRP compared with bmMSC.

Bone Marrow Aspirate Concentrate-PRP on Partial Tear of the Rotator Cuff Tendon

Nonsurgical treatment for rotator cuff tendon tears includes exercise, medication, and corticosteroid injections. These approaches provide stable long-term outcomes, including but not limited to pain and symptomatic relief, increased QOL, and faster return to work. Regenerative bmMSC biological treatment and PRP were compared to a control group treated with physical therapy techniques (i.e., scapular stretching, stabilization, and strengthening) to assess the improvement of symptoms in those with rotator cuff tears. Over the course of three months, the researchers found that pain and shoulder function improved after the injection of bone marrow aspirate concentrate (BMAC)-PRP compared to the control group [31]. In this study, the researchers did not perform individual tests on the effect of BMAC versus PRP due to the procedure being invasive and the desire to develop a treatment that demonstrated a maximal therapeutic effect. Tear size failed to improve, as it is dependent on surgical intervention [31]. From these results, decreased pain can be potentially attributed to the decreased inflammatory mediators as a result of treatment with PRP and BMAC [31]. Although this study only maximized therapeutic benefits such as pain reduction, PRP and BMAC should be further investigated as a treatment option using a broader range of participants to determine the presence of side effects and the total therapeutic benefit.

BMAC-PRP on Tendon-Derived Stem Cells and Rotator Cuff Tendon Tear

Kim et al. focused on the effects of combining PRP with BMAC to determine the effects of this combination on rotator cuff tendon repair. The results of this study show that dual therapy with PRP plus BMAC resulted in decreased partial tear size (initial: 30.2$\pm$24.5 mm^2^ to 22.5$\pm$18.9 mm^2^ at 3 months with a p>0.05) and an increase in growth and migration of tendon-derived stem cells, while preventing aberrant chondrogenic and osteogenic differentiation [32]. A VAS score of 5.8$\pm$1.9 in the PRP plus BMAC pretreatment decreased to 2.8$\pm$2.3 at 3 months after treatment with PRP plus BMAC which demonstrated a reduction in clinical symptoms. It was shown that there is a specific stem cell differentiation induced with treating with a dual therapy increasing the accuracy and target of treatment; thus, dual therapy should be further considered for rotator cuff tendon repair over current arthroscopy and open surgery treatment, which hold the risk of fatal complications such as stroke, heart attack, pneumonia, or blood clots [32]. The dual therapy approach could offer a more advanced framework for treating the underlying pathology because the PRP provides a growth factor scaffold potentially aiding the regenerative potentials of BMAC [32]. Due to the limitations in this study of not having a control group to compare with the experimental group, dual therapy should be further considered, studied, and analyzed for long-term benefit and complications in comparison to both arthroscopic and open surgical approaches.

Discussion

Overall, multiple studies concluded the necessity for future research on the usage and effects of PRP and stem cells in the treatment of MSK-related injuries. Both types of injections allow for an increase in regeneration ability using endogenous material from each patient, a less invasive approach, lower side effects, and potential healing for the various parts of the MSK system. Some of the risks involved in PRP and stem cell injections include immune reactions such as inflammation and infections from the injections. But current studies have found very few side effects and the injections to be relatively safe [33-35]. However, further research is required on the risk factors that PRP and stem cell injections may pose. Another aspect to be considered is the cost of both injections. Both stem cells and PRP injections are not covered by many health insurance providers, leading to their high cost. According to Fuggle et al., the cost for a single stem cell injection for OA could be on average $5,156 and for a PRP injection about $714 [35]. Therefore, other treatment options such as steroid injections are favored due to the lower cost per injection. A common limitation throughout the studies was a lack of standardized protocol with creating the PRP and stem cell injections, which leads to the difficulty of comparing the effectiveness of multiple studies effectiveness between one another. No consistent standardized protocol creates a challenge of determining the effectiveness of each injection for the specific injuries acquired. Due to the varying results in the literature on a range of conditions, a broad generalization that PRP and/or stem cell injections are universally effective is not possible. Therefore, in the future, a standardized protocol for the creation of PRP and stem cell injections is needed in order to better compare the results of each study to the other.

## Conclusions

This review outlined the current state of research evaluating PRP and stem cell injections in the treatment of various MSK injuries. Both approaches have shown the potential to improve an individual’s QOL, maintain long- term efficacy, and are associated with fewer side effects as compared to standards of care in multiple MSK injuries such as plantar fasciitis, Achilles tendinopathy, acute muscle and tendon tears, ligament injuries, chondral and MCL knee injuries and arthritis, rotator cuff lesions, and avascular femoral necrosis. PRP and stem cells can provide an alternative solution to common MSK-related injuries by decreasing the requirement for pain medication, decreasing adverse effects, and complications associated with more invasive procedures. Meanwhile, a limitation of both PRP and stem cell therapies is the lack of a standardized protocol to objectively compare the results of multiple studies together. It is important to note that current research is ongoing and new experiments, clinical studies, and results are continuously evolving to meet the demand for understanding this increasingly complex and popular therapy. As such, further research is still needed in order to explore their efficacy and safety in larger, randomized controlled trials.
